# Enhancing task-demands disrupts learning but enhances transfer gains in short-term task-switching training

**DOI:** 10.1007/s00426-020-01335-y

**Published:** 2020-04-18

**Authors:** Katrina Sabah, Thomas Dolk, Nachshon Meiran, Gesine Dreisbach

**Affiliations:** 1 Department for Experimental Psychology, Regensburg University, Universitätstraße 31, 93053 Regensburg, Germany; 2 Ben Gurion Univsersity of the Negev, Beersheba, Israel

## Abstract

Content variability was previously suggested to promote stronger learning effects in cognitive training whereas less variability incurred transfer costs (Sabah et al. Psychological Research, 10.1007/s00426-018-1006-7, 2018). Here, we expanded these findings by additionally examining the role of learners’ control in short-term task-switching training by comparing voluntary task-switching to a yoked control forced task-switching condition. To this end, four training conditions were compared: (1) forced fixed content, (2) voluntary fixed content, (3) forced varied content, and (3) voluntary varied content. To further enhance task demands, bivalent stimuli were used during training. Participants completed baseline assessment commencing with task-switching and verbal fluency blocks, followed by seven training blocks and last by task-switching (near transfer) and verbal fluency (far transfer) blocks, respectively. For the baseline and transfer task-switching blocks, we used the exact same baseline and first transfer block from Sabah et al. (Psychological Research, 10.1007/s00426-018-1006-7, 2018), employing univalent stimuli and alternating-runs task sequence. Our results pointed again to the contribution of content variability to task-switching performance. No indications for far transfer were observed. Allowing for learners’ control was not found to produce additional transfer gains beyond content variability. A between-study comparison suggests that enhanced task demands, by means of bivalency, promoted higher transfer gains in the current study when compared to Sabah et al. (Psychological Research, 10.1007/s00426-018-1006-7, 2018). Taken together, the current results provide further evidence to the beneficial impact of variability on training outcomes. The lack of modulatory effect for learners’ control is discussed in relation to possible methodological limitations.

**Electronic supplementary material:**

The online version of this article (10.1007/s00426-020-01335-y) contains supplementary material, which is available to authorized users.

## Introduction

Cognitive or “brain” training has evoked a heated debate regarding its effectiveness in inducing compelling and generalizable improvements in cognitive functions. Recent meta-analyses show that there is no strong evidence for the transferability of training-related benefits to structurally different tasks (i.e., far transfer) or real-life situations (e.g., Doughertyet al. 2016; Melby-Lervåg et al. 2016; Soveri et al. 2017). Nonetheless, consistent results support the occurrence of transfer to novel structurally similar tasks (i.e., near transfer; Karbach and Verhaeghen 2014; Schwaighofer et al. 2015). Consequently, and given the significant clinical and social implications of cognitive interventions, it seems warranted at this point to step back to reflect upon and examine the underlying mechanisms for Cognitive Training (CT) effectiveness. For example, recent attempts have introduced the notion of variability as a possible moderator for learning generalization (e.g., Karbach and Kray 2009; Sabah et al. 2018). Findings indicate that the so far undertaken approach in CT studies of *doing more of the same* (i.e., task repetitiveness), seems to have transfer costs. That is, repetitive practices actually seem to perpetuate rigid behavioral patterns (Sabah et al. 2018).

Specifically, Sabah et al. (2018) observed that manipulating content variability in short-term task switching training (tasks and stimuli either changed in every block or remained the same throughout the training phase) counteracted the potentially deteriorating effects of repetitive training. Interestingly, a dissociation between learning and transfer has been revealed: Participants who practiced the same two tasks throughout training (i.e., fixed content condition) showed a steep learning curve but also showed significant transfer *costs* when confronted with two new tasks. Conversely, participants who received varying training tasks (i.e., varied content condition) showed a much flatter learning curve but critically smaller transfer *benefits* rather than costs. As such, we concluded that (1) training benefits are not a valid proxy for successful transfer, and (2) increased task demands during training prevent transfer costs. Here we aim to expand this line of research: First, we drew on these prior results as well as on the possible added value of higher task interference demands during training for transfer (Schmidt and Bjork 1992). That is, unlike our previous study, in which univalent stimuli were used (a given stimulus was unequivocally associated with only one task), here we aimed to increase between task interference and thus task demands by always presenting two stimuli, one of each ask on a given trial. The idea is that this presentation mode would require top-down cognitive control, namely, knowing which task is currently required and would thus increase task engagement and reliance on cognitive control processes.

Moreover, and for the first time, we aimed at examining another possible moderator for determining the efficacy of cognitive training outcomes: *learners’ control*. While so far the literature on cognitive training has focused on external features pertaining mostly to training design, less focus has been given to intrinsic features related to the trainee himself, such as motivation, cognitive abilities, beliefs, expectancies, and self-generated goals. Surprisingly, some of these factors like motivation have rather been treated as an undesirable confound (e.g., Jolles et al. 2012; Morrison and Chein 2011). This state of affairs is surprising given the empirical evidence and theoretical models linking the aforementioned internal states and individual differences to learning and transfer (e.g., Ackerman 1987; Baldwin and Ford 1988; Bürki et al. 2014; Quiñones 1995; Ruona et al. 2002). We thus strived to investigate whether granting trainees control over their practice schedule would benefit learning. To this end, we used a task-switching paradigm, allowing us to manipulate training variability in terms of content (i.e., stimuli and task rules) as well as trainees’ control over the task sequence. Training variability was achieved by new task rules and stimuli in each training block (as compared to repeating the same task rules throughout the blocks) as in Sabah et al. (2018). Trainees’ control was manipulated by comparing the more standard instructed task-switching paradigm with the *voluntary* task-switching paradigm where participants have to choose themselves which of two available tasks to perform on each trial (Arrington and Logan 2004, 2005).

## Passing the torch: considering learners’ role in cognitive training

The recognition that a learner is more than just a passive recipient but rather an active agent has long been central to learning and cognitive theories such as constructivism theories, cognitive flexibility theory and multiple intelligences theory (e.g., Gardner 1987; Piaget 1980; Spiro and Jehng 1990). These ideas have unsurprisingly inspired many instructional approaches (e.g., Bell and Kozlowski 2008; Chiviacowsky et al. 2012a, b; Mayer and Moreno 2003).

Despite the remarkable body of literature on learners’ control giving evidence to its contribution to learning outcomes across domains, the topic in the realm of CT remains underappreciated. This is quite intriguing when considering the fact that the most promising training outcomes with wider transfer effects are attributed to video gaming training (Al-Hashimi et al. 2013; Colzato et al. 2010; Green and Bavelier 2003; Olfers and Band 2018). After all, aside from the favorable environmental variability embedded within video gaming platforms, learners’ control might also play an additional and critical component contributing to the beneficial training outcomes.

Why should learners’ control be beneficial for cognitive training in the first place? In our understanding, possible benefits are grounded mainly on CT’s interlink to the notion of desired difficulty—highlighting the paradox of mental effort (Bjork 1994; Dougherty et al. 2016; Inzlicht et al. 2018; Schmidt and Bjork 1992). According to this paradox, while engagement in highly demanding cognitive tasks appears to be costly and aversive, a certain amount of difficulty is actually desirable and promotes better long-term learning outcomes (Bjork and Bjork 2011; Healy et al. 2014; Schneider et al. 2002). This in turn seems very relevant to CT when considering the costly and aversive nature of cognitive effort, markedly manifested in training protocols, such as those targeting highly demanding processes of working memory, inhibition of automatic tendencies and switching between tasks (Braver 2012; Kool et al. 2010; Monsell 2003; Westbrook et al. 2013). Hence, it is postulated that learners’ control might allow individualized, strategic and flexible adaptation of effort allocation. This prevents depletion while at the same time maintaining a desired level of difficulty to avoid boredom, thereby allowing learning to occur (e.g., Ackerman 1987; Inzlicht et al. 2014; Kinzie 1990; Muraven et al. 2006; Navon and Gopher 1979; Paas et al. 2005). Similarly, it has been suggested that deliberate and self-initiated practice rather than merely repetitive extended practice underlies expert behavior (e.g., Ericsson et al. 1993). This claim is based on the observation that skill acquisition requires by itself only limited amount of practice with individuals reaching a performance asymptote quite rapidly without additional improvement hereafter (i.e., automaticity; Anderson 1982; Fitts and Posner 1967). In contrast, the “deliberate practice” framework emphasizes the role of high motivation for seeking demanding tasks as well as engagement in self-monitoring processes (e.g., error identification and correction) to overcoming automaticity and supporting progressive learning and improvement (Ericsson 2006, 2008).

## Should I stay or should I switch: considering voluntary task-switching in training cognitive flexibility

Task-switching ability, widely considered as a marker of cognitive flexibility, is measured by the costs incurred in response times and accuracy when switching as compared to repeating cognitive tasks (for reviews, see Kiesel et al. 2010; Monsell 2003; Vandierendonck et al. 2010). Task-switching has also become central to cognitive enhancement studies (e.g., Karbach and Kray 2009; Karbach et al. 2010; Kray and Fehér 2017; Minear and Shah 2008). One advantage of the Task-switching paradigm is that it can help us gain a better understanding of the variables moderating training outcomes, such as training variability (Karbach and Kray 2009; Minear and Shah 2008; Sabah et al. 2018). Moreover, it allows to investigate an important additional moderator, learners’ control. To allow for this, the voluntary task-switching paradigm was used, enabling participants to voluntarily choose on any given trial which task they want to perform (Arrington and Logan 2004). Moreover, given that participants themselves have to decide which task to execute on any given trial, the VTS paradigm engages participants in goal setting and thus in a relatively more active self- regulated processing (Arrington and Logan 2004), which is arguably integral to learning and transfer.

As stated above, in our previous study (Sabah et al. 2018), content variability in task switching was shown to undermine the costly outcomes of repetitive training. Despite the observed improvement in task switching performance across the learning blocks, participants in the fixed content condition, produced transfer costs. In contrast, participants in the varied content condition seemed to have benefitted from content variability, however in the absence of improvement during learning. From here, this dissociation between learning and transfer performance falls in line with previous suggestions that advocate “desired difficulty” manipulations, such as content variability, to promote better learning generalization (Schmidt and Bjork 1992).

Expanding our previous line of research, we strived to examine here whether (a) additional benefits would arise when increasing between task interference during training and allowing for learners’ control in the varied content condition and, (b) whether negative transfer costs would be prevented in the fixed content condition by the same means (increased interference, allowing for learners’ control). To this end, we ran a CT-task-switching study and manipulated content variability and whether the tasks were voluntarily chosen. We thus compared four conditions: (a) voluntary varied content (voluntary VC), (b) forced varied content (forced VC), and (c) voluntary fixed content (voluntary FC), and (d) forced fixed content (forced FC). For the forced conditions, a yoked control procedure was followed.^1^ Specifically, pre-post Task-switching performance was examined (near transfer measure), introducing untrained task stimuli and rules as well as a distinct task sequence (alternating runs, e.g., with Tasks A and B the sequence was AA–BB–AA…) on both the baseline and transfer blocks. Similarly, far transfer effects were examined using the verbal fluency task, a measure of cognitive flexibility that has a switching element (e.g., Troyer et al. 1997; Troyer et al. 1998). Specifically, we predicted the following:

1. For content variability manipulation, we expected to replicate and extend our previous findings, pointing to the advantage of content variability training over fixed content condition, reflected in better task-switching performance in near transfer coupled with less improvement during training (Sabah et al. 2018). The novelty here is that we also examined whether there would be far transfer effects seen in verbal fluency.
2. Allowing learners’ control in Task-switching training was predicted to promote additional transfer benefits in the voluntary VC when compared to forced VC condition. Additionally, we aimed to explore whether the increased task demands due to increased task interference during training (bivalency) would diminish the transfer-costs in Task-switching performance which we observed before (Sabah et al. 2018) in the fixed-content group. To this end, the results of the current study will be compared to those obtained by Sabah et al. (2018).

Finally, even though not being part of our main question, the design allows to explore training effects on the voluntary switch rate as a function of varied vs. fixed content. Given the evidence for bottom-up (e.g. Mayr and Bell 2006) and context effects (Fröber and Dreisbach 2017) on voluntary task-switching, one may predict higher switch rates when stimuli and tasks change in every block. On the other hand, given the literature on motivation and boredom one could predict the opposite, namely increased switch rates when stimuli and tasks never change (Inzlicht et al. 2014).

## Methods

### Participants

One hundred and sixty Regensburg University students (16 males; *M*_age_ = 22.9, 95% CI [22, 23]) were compensated with either one-hour course credit (*n* = 21) or were paid 6€ (6.89$; *n* = 99). All participants reported having normal or corrected-to-normal vision and gave written consent prior to their participation in the study.

### Apparatus

All experimental tasks were programed in E-prime (Psychology Software Tools, Pittsburgh, PA, USA). The experiment was controlled by Dell computer with 19ʺ flat screen.

#### Verbal fluency task (baseline and transfer)

Stimulus presentation and response recording were computerized. Vocal responses were collected using an external voice recorder (TASCAM linear PCM recorder DR-05). The test began with a general instruction slide, which was followed by two test blocks. Participants were instructed to produce as many words that start with the target letter in 60 s while avoiding proper names, same stem words, verb conjunctions or numbers. Participants were presented with two letters (either F, B or N, T), one in each block. We counterbalanced (between participants) the assignment of letter-pair to blocks (e.g., whether F–B were presented in Block 1 and N–T in Block 2 or vice versa) and also counterbalanced the order of letters (e.g., FB or BF) within the block. (It is worth noting that the chosen letters were validated in German where each pair consisted of one hard and one easy item; see Schmidt et al. 2017). Each test block started with a screen asking participants to press the space-bar button when ready to perform the first task, followed by a visual and auditory presentation of the target letter for 1000 ms. A presentation of a one-minute sand clock followed, indicating the remaining time.

#### Task-switching (baseline, transfer and training)

In total, nine different task pairs were employed, each composed of two task rules, one for pictures and one for words stimuli (See Table 1). The pictorial stimuli were sized 1.57″ × 1.18″ whereas the word stimuli were printed in 30px Calibri Light font. For each task rule, eight exclusive stimuli were used (four stimuli for each category) that were assigned to either a left response key (z or n) or right response key (x or m) on a QWERTZ-keyboard, depending on the respective category. The response key assignment to a given category was counterbalanced across participants. A modified version of the task-switching paradigm was used, including solely mixed blocks.

##### Baseline and transfer

In baseline and transfer we used a predictable task order of alternating runs (task sequence Picture-Picture-Word-Word… etc.), with univalent stimuli (when the task involved a picture, only a picture was presented, and when it involved a word, only a word was presented). We used the exact same baseline (Pair A) and transfer (Pair I) tasks as in Sabah et al (2018).

##### Training

The stimuli were bivalent (involving the simultaneous presentation of a picture and a word). For a given participant, stimuli pertaining to the one rule were constantly presented above and stimuli pertaining to the other rule were presented below the fixation cross (counterbalanced across participants). Whether the two tasks remained the same throughout training and whether participants could choose the tasks, was determined by the experimental group (see general procedure).

In all, baseline, transfer and training, each task-switching block started with two instructional slides presenting the task rules, followed by eight practice trials. Thereafter, a block of 64 experimental trials started, ending with a feedback slide presenting the statistics for this block including percentage of correct responses, mean reaction time, and switch rate (in the voluntary group). In each trial, participants were asked to classify picture stimuli (Task Rule 1) or word stimuli (Task Rule 2) to a corresponding rule. In the voluntary conditions, participants chose which task rule to execute. In the forced conditions, the required task rule (Picture/Word) was indicated by placing a rectangle around the relevant target stimulus. Stimuli remained on screen either until a response was given or until 3500 ms had elapsed. After an inter-stimulus interval of 500 ms the next trial started. Feedback was only presented for errors or too slow reaction times (slower than 3500 ms).

**Table 1:** Task rules used in the training and transfer blocks

Pair	Task Rule 1 (Pictorial)	Task Rule 2 (Words)
Pair A (Baseline, the same for all groups)	Is it summer or winter related?	Is it a man’s or woman’s name?
Pair B	Is it sea or land transportation?	Can it be seen or heard?
Pair C	Is it vegetable or fruit?	Is it black or white material?
Pair D	Is it shoes or body parts?	Is it alcoholic or non-alcoholic drink?
Pair E	Is it mammalian or bird?	Is it old or new invention?
Pair F	Is it a cat or dog?	Is it located in Asia or Europa?
Pair G	Is it clothes or furniture?	Is it sweet or salty?
Pair H	Is it an electronic device or road sign?	Is it a hot or a cold meal?
Pair I (same for all groups)	Is it a musical or a sports instrument?	Is it a flower or tree?

The order of pairs B–H was counterbalanced across participants. In the fixed content groups, Pair A from baseline was also used in all experimental blocks. Pair I was the same for all groups

### General procedure

Participants were randomly assigned to one of four equal sized groups: (a) voluntary VC, (b) forced VC, (c) voluntary FC, and (d) forced FC. They attended a one-hour experimental session, starting with baseline: verbal fluency test block, followed by one baseline task-switching block (with univalent targets). Training consisted of seven task-switching blocks with bivalent targets. In the task-switching training blocks, participants in the VC conditions received two new task rules on each block whereas the same two task rules were performed across all blocks in the FC conditions (i.e., Pair A). In the voluntary switching conditions, participants were asked to freely choose which task to perform on a given trial, with the restriction to perform each task equally often and in random order as if “flipping a coin” (cf. Arrington and Logan 2004). In the forced switching conditions, each participant was yoked to one of the individuals in either the voluntary FC or VC condition depending on group assignment, so it matched in task selection on each corresponding trial and switch rate. The session ended with one task-switching transfer block (univalent targets) followed by a verbal fluency transfer block.

## Results: task-switching

Data analysis was conducted following the protocol of our previous study (Sabah et al. 2018) to maintain a high comparability between the two studies. Thus, following the same exclusion criteria, participants with excessive error rates (above 20% as compared to 4% in the remaining sample) in either the task-switching baseline or transfer blocks were excluded from the analysis. Consequently, data from two participants in the forced FC group were discarded.

For response time (RT) analysis, practice trials, erroneous trials, trials following an error as well as the first experimental trial of each block were discarded (11%). For mean error rates, see Figure A1 and Figure A2 in Appendix A.

In addition, Bayes Factor (BF) analyses were carried out using JASP (JASP team, Version 0.11.1.0), contrasting H0 (no effect) with H1 which was specified using the default priors. We report BF_10_ (advantage of H1 over H0) and BF_01_ (advantage of H0 over H1). BF_10_ for 2-way interaction effects was computed by dividing the BF_10_ of a model with main effects and interaction by the BF_10_ of a (respective) main-effect-only model. Similarly, BF_10_ for triple interactions was computed by dividing the BF_10_ of a full model including all the main effects, 2-way interactions and the triple interaction by the BF_10_ of a similar model not including the triple interaction.

### Initial differences between the groups

To look for potential initial differences between the training groups, 4 (Group: voluntary VC, forced VC and voluntary FC and forced FC, between participants) × 2 (Trial Type: repeat, switch, within participants) Frequentists and Bayesian analysis of variance (ANOVAs) were conducted on (a) RTs and (b) error rates of the baseline block.

#### RTs

The results revealed the typical switch cost pattern (*M*_repeat_ = 612 ms, 95% CI [597, 628]; *M*_switch_ = 764 ms, 95% CI [737, 791]), *F*(1,154) = 245.64, *p* < 0.001, *η*_p_^2^ = 0.62, BF_10_ > 100, whereas no significant difference was found between groups, *F*(3,154) = 0.48, *p* = 0.69, *η*_p_^2^ = 0.01, BF_10_ = 0.05. Additionally, group did not interact with trial type, *F*(3,154) = 1.04, *p* = 0.37, *η*_p_^2^ = 0.02, BF_10_ = 0.12.

#### Error rates

Error rates were generally low (*M* = 0.04; 95% CI [0.03, 0.04]). The typical switch cost pattern was also revealed in error rates, with participants making more errors on switch (*M*_switch_ = 0.04, 95% CI [0.04, 0.05]) when compared to repeat trials (*M*_repeat_ = 0.03, 95% CI [0.02, 0.03]), *F*(1,154) = 20.05, *p* < 0.001, *η*_p_^2^ = 0.11, BF_10_ > 100. Neither the main effect for group, *F*(3,154) = 0.19, *p* = 0.90, *η*_p_^2^ = 0.004, BF_10_ = *0.0*6, nor the interaction between group and trial type reached significance, *F*(3,154) = 1.28, *p* = 0.28, *η*_p_^2^ = 0.02, BF_10_ = 0.07.

### Training performance (block 1–7)

**Fig. 1:**
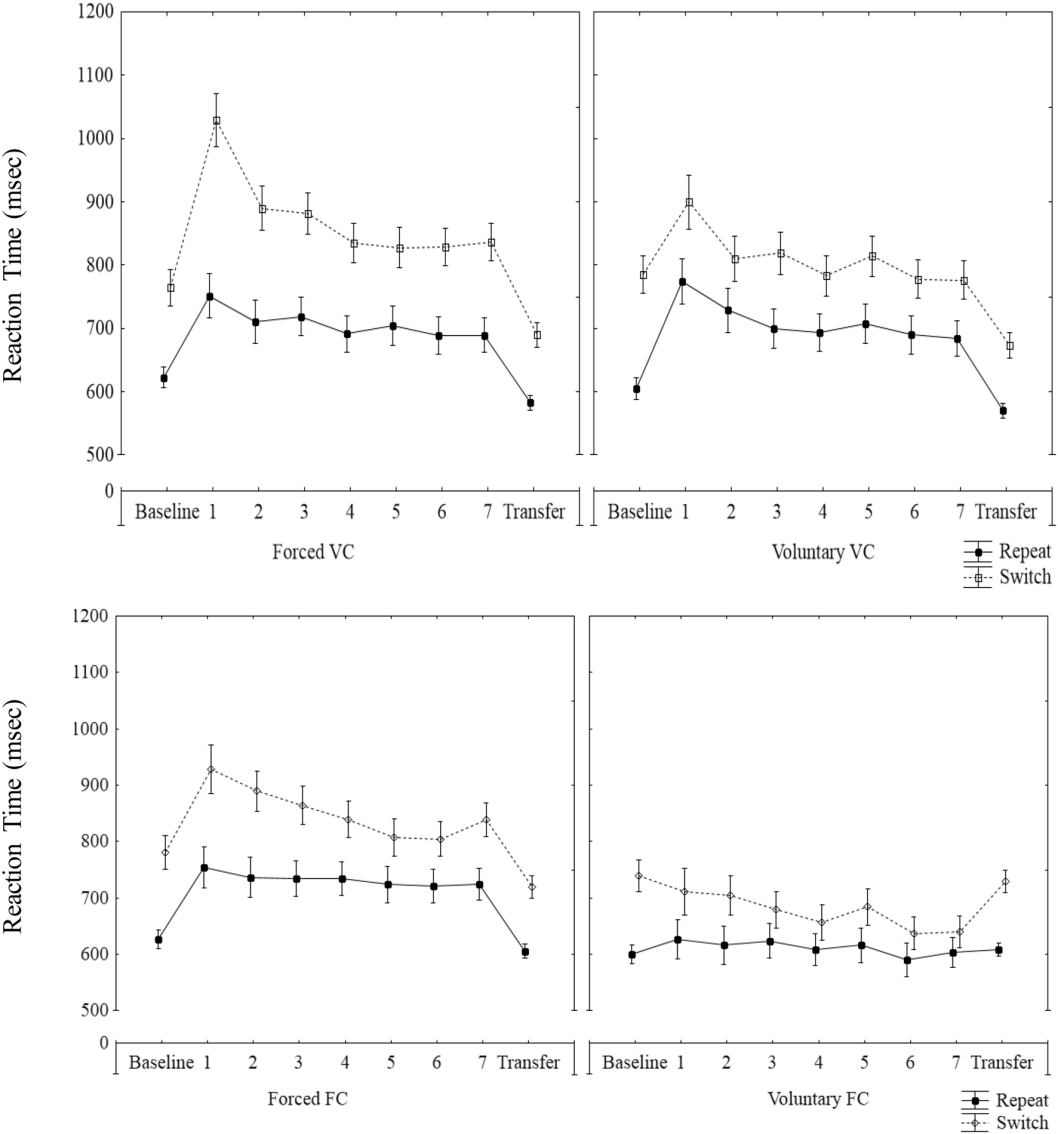
Mean reaction time (RT) in ms as a function of trial type across the experimental blocks in the groups. Error bars represent standard error of the mean

To analyze training performance, a four-way Frequentists and Bayesian ANOVA was performed with content (fixed vs. varied) and learners’ control (Forced vs. VTS) as between-subject variables and block (1–7) and trial type (repeat, switch) as within-subject variables for both RTs and error data (see ANOVA Tables 2 and 3, respectively). Figure 1 presents mean RTs over all (training and transfer) blocks in the four training groups.

#### RTs

Statistics are depicted in Table 2. The results reveal a significant main effect for block, pointing to generally decreasing RTs from block 1 to block 7 (*M*_Block1_ = 809 ms, 95% CI [773, 846]) vs. *M*_Block8_ = 724 ms, 95% CI [698, 751]). Likewise, a significant main effect was obtained for trial type, showing the typical switch costs (*M*_Repea*t*_ = 691 ms, 95% CI [663, 719]; *M*_Switc*h*_ = 803 ms, 95% CI [774, 833]). A significant main effect was found for content, where faster RTs were observed in the FC (*M* = 718 ms, 95% CI [678, 757]) when compared to the varied content condition (*M* = 776 ms, 95% CI [737, 816]). However, the Bayes Factor (BF) for content was inconclusive (i.e., representing “anecdotal evidence” for the alternative hypothesis). The main effect for learners’ control also reached significance with participants being slower in the forced (*M* = 792 ms, 95% CI [753, 832]) as compared to the voluntary task-switching condition (*M* = 702 ms, 95% CI [663, 741]).

The interaction between trial type and block was significant, pointing to decreasing switch costs along the course of training: Significantly lower switch costs were observed in the last training block (Block 7; *M* = 87, 95% CI [68, 107]) when compared to the first training block (*M* = 163, 95% CI [136, 189]), *t*(151) = 5.07, *p* < 0.001, *d* = 0.41. Likewise, the interaction between content and trial type reached significance, with switch costs being higher in the VC (*M* = 134, 95% CI [114, 155]), when compared to FC groups (*M* = 89, 95% CI [69, 109]), *t*(156) = 3.72, *p* < 0.001, *d* = 0.59. Strong evidence for the alternative hypothesis is confirmed by the BF for the interactions of Trial type × Block and Content × Trial type.

The interaction between content and block was significant. An improvement in RTs was noted among both the FC and VC conditions, *t*(77) = 4.20, *p* < 0.001, *d* = 0.40, *t*(79) = 5.11, *p* < 0.001, *d* = 0.57, respectively. Participants in the VC condition, showed slower RTs in both the first and last training block, *t*(156) = 2.8, *p* < 0.01, *d* = 0.23, *t*(156) = 2.01, *p* < 0.05, *d* = 0.32, respectively. Also, a significant interaction between content and learners’ control was found. In the VC condition, no significant difference in RTs was found between the forced and voluntary task-switching condition, *F*(1,71) = 0.63, *p* = 0.43, *η*_p_^2^ = 0.01. In contrast, in the FC condition, significantly slower RTs were observed in the forced when compared to the voluntary task-switching condition, *F*(1,71) = 13.09, *p* < 0.01, *η*_p_^2^ = 0.16. The two-way interactions Content × Block and Content × Learners’ control should, however, be interpreted with caution, because the corresponding BF did not indicate sufficiently strong evidence.

**Fig. 2:**
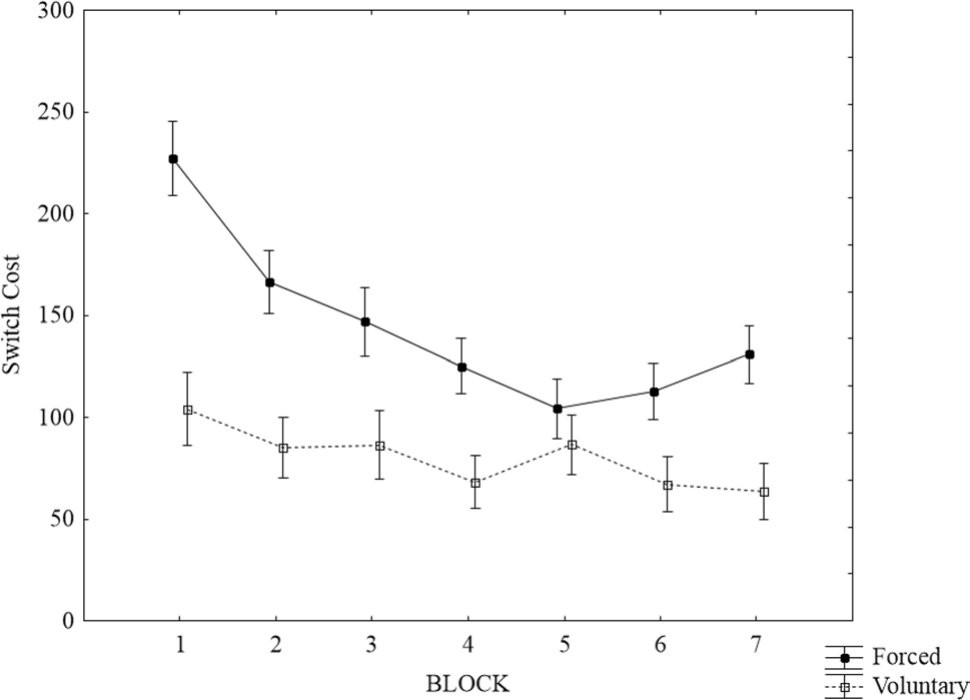
Mean switch cost (in msec) as a function of Block and Condition (forced vs. voluntary) collapsed across content. Error bars represent standard error of the mean

Moreover, the two-way interaction between learners’ control and trial type was further qualified by higher-order interaction between learners’ control, trial type and block. As shown in Fig. 2, in the first training block, higher switch costs were observed in the forced vs. voluntary task-switching condition, *t*(150) = 4.87, *p* < 0.001, *d* = 0.79. While participants in the forced condition showed a decrease in switch costs with increasing training, no distinct pattern was found within the voluntary task-switching condition. Significant reductions in switch costs between the first and last training blocks were observed in both the forced and voluntary task-switching conditions, *t*(74) = 4.74, *p* < 0.001, *d* = 0.55; *t*(76) = 2.39, *p* < 0.05; *d* = 0.27, respectively. In the last training block, significantly higher switch costs were obtained in the forced as compared to the voluntary task-switching condition, *t*(148.16) = 3.14, *p* < 0.001, *d* = 0.50. The Bayesian analysis results were not aligned with the frequentist statistics, with BF suggesting a strong evidence for the two-way interaction Learners’ control × Trial type but not for the three-way interaction Leaners’ control × Trial type × Block.

The three way-interactions Content × Learners’ control × Trial type and Content × Trial type × Block as well as the four-way interaction Content × Learners’ control × Trial type × Block did not reach significance. The corresponding Bayesian analyses provided strong evidence for the null hypothesis.

Taken together, fixed content incurred smaller switch costs than varied content. In addition, switch costs were smaller in the voluntary task-switching conditions as compared to the forced switching conditions. A notable reduction in switch costs along the training blocks was observed solely in the forced condition. However, this latter effect was very small and not confirmed by the Bayesian analysis. Likewise, the interaction Block × Content replicated previous findings (Sabah et al. 2018) in showing steeper learning in the FC as compared to the VC groups. Note, though, that the effect was numerically small and its presence was not confirmed by the Bayesian analysis.

**Table 2:** Main effects and interaction of the Content ×learners’ Control × Block (1–7) × Trial type ANOVA (RTs)

	Statistic	*p* value	Effect size (*η*_p_^2^*)*	BF_10_
Content	*F*(1, 142) = 4.34	< 0.05^*^	0.03	1.60
Learners’ control	*F*(1, 142) = 10.23	< 0.01^**^	0.07	17.50
Block	*F*(6, 852) = 22.34	< 0.001^***^	0.14	> 100
Trial type	*F*(1, 142) = 273.96	< 0.001^***^	0.66	> 100
Content × Learners’ control	*F*(1, 142) = 4.43	< 0.05^*^	0.03	2.35
Trial type × Block	*F*(6, 852) = 9.23	< 0.001^***^	0.13	59
Content × Block	*F*(6, 852) = 2.935	< 0.01^**^	0.02	0.22
Learners’ control × Block	*F*(6, 852) = 1.41	0.21	0.01	< 0.01
Content × Trial type	*F*(1, 142) = 10.18	< 0.01^**^	0.07	> 100
Learners’ control × Trial type	*F*(1, 142) = 21.82	< 0.001^***^	0.13	> 100
Content × Learners’ control × Block	*F*(6, 852) = 0.10	0.99	0.001	< 0.01
Content × Learners’ control × Trial type	*F*(1, 142) = 0.09	0.77	0.001	0.20
Content × Trial type × Block	*F*(6, 852) = 1.10	0.40	0.01	< 0.01
Learners’ control × Trial type × Block	*F*(6, 852) = 3.40	< 0.01^**^	0.02	0.08
Content × Learners’ control × Trial type × Block	*F*(6, 852) = 0.86	0.53	0.06	< 0.01

#### Error rates

Overall, error rates were low (*M* = 0.04; 95% CI [0.04, 0.05]). As shown by Table 3, the main effect for trial type reached significance. Participants made slightly more errors on switch (*M* = 0.05; 95% CI [0.04, 0.05]) when compared to repeat trials (*M* = 0.04; 95% CI [0.04, 0.05]). The interaction Block x Content was also significant. On the last training block, participants in the VC condition made less errors (*M* = 0.03; 95% CI [0.03, 0.04]) when compared to the FC condition (*M* = 0.05; 95% CI [0.04, 0.05]), *t*(156) = 2.55, *p* < 0.05, *d* = 0.56. Neither the BF for the main effect trial type nor for the interaction Block × Content suggested strong evidence for the alternative hypothesis. All other effects did not reach significance with all corresponding BF_10_ values providing strong evidence for the null hypothesis.

**Table 3:** Main effects and interaction of the Content × Learners’ control × Block (1–7) × Trial type ANOVA (error rates)

	Statistic	*p* value	Effect size (*η*_p_^2^*)*	BF_10_
Content	*F*(1, 146) = 0.28	0.60	0.001	0.12
Learners’ control	*F*(1, 146) = 0.14	0.71	0.07	0.11
Block	*F*(6, 876) = 1.57	0.15	0.01	0.004
Trial type	*F*(1, 146) = 4.52	< 0.05^*^	0.03	0.64
Trial type × Block	*F*(6, 876) = 0.53	0.78	0.004	< 0.01
Content × Block	*F*(6, 876) = 2.34	< 0.05^*^	0.02	< 0.01
Learners’ control × Block	*F*(6, 876) = 1.26	0.27	0.01	< 0.01
Content × Trial type	*F*(1, 146) = 0.01	0.93	0.001	0.07
Learners’ control × Trial type	*F*(1, 146) = 0.03	0.85	0.001	< 0.01
Content × Learners’ control × Block	*F*(6, 876) = 0.59	.74	.004	< 0.01
Content × Learners’ control × Trial type	*F*(1, 146) = 2.69	0.10	0.02	< 0.01
Content × Trial type × Block	*F*(6, 876) = 1.39	0.21	0.01	< 0.01
Learners’ control × Trial type × Block	*F*(6, 876) = 0.99	0.43	0.01	< 0.01
Content × Learners’ control × Trial type × Block	*F*(6, 876) = 0.57	0.76	0.003	< 0.01

#### Voluntary switch rate

**Fig. 3:**
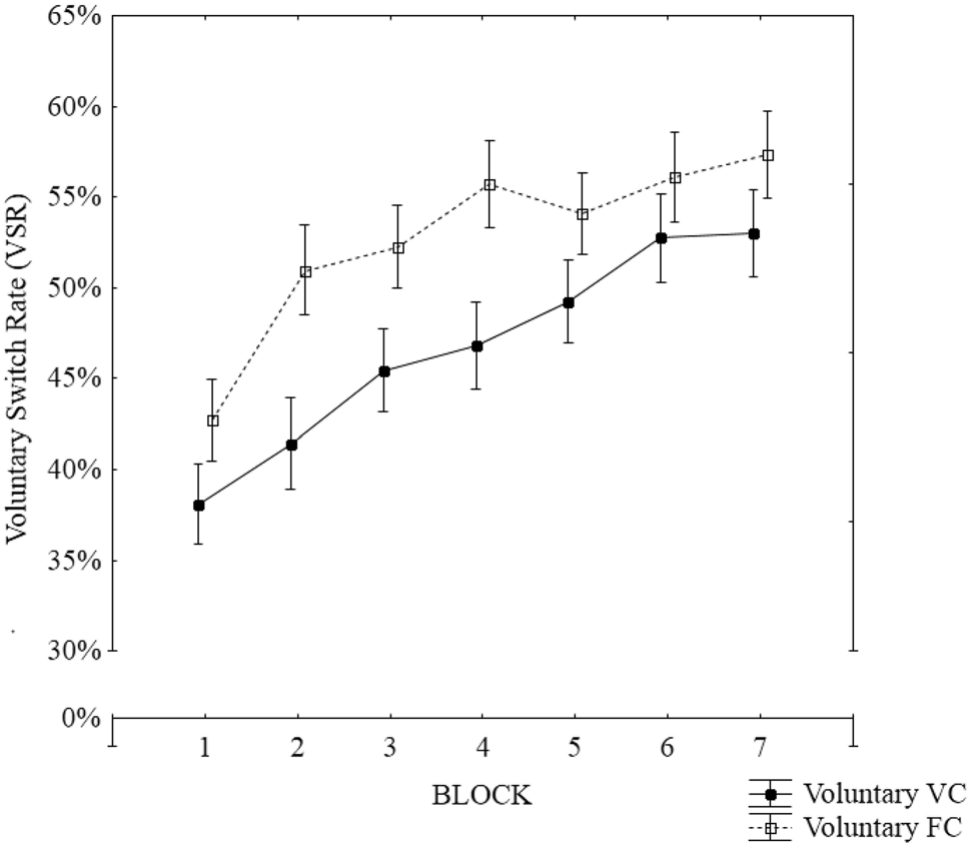
Mean switch rates in the voluntary FC and VC conditions along the training blocks. Error bars represent standard error of the mean

To examine possible differential learning features, switching rates in the VS groups were analyzed. To capture all attempted switches, erroneous trials were also included (cf. Arrington and Logan 2004). To this end, a 2 × 7 mixed model Frequentists and Bayesian ANOVA were conducted with group as a between-subject variable (voluntary VC and voluntary FC) and block (block 1–7) as within-subject variable. The results brought up a significant main effect for group, pointing to higher switch rates (*M* = 53, 95% CI [49, 56]) in the FC group when compared to the voluntary VC group (*M* = 47, 95% CI [43, 50]), *F*(1,78) = 6.66, *p* < 0.05, *η*_p_^2^ = *0.0*8, F(6, 468) = 16.06, *p* < 0.01, *η*_p_^2^ = *0.1*7, BF_10_ = 3.622, respectively. In addition, the main effect for block reached significance, reflecting the continuous increase in switch rate with increasing training, *F*(6, 468) = 16.06, *p* < 0.01, *η*_p_^2^ = *0.1*7, BF_10_ > *100* (see Fig. 3). The interaction between block and content was not significant (*F* < 1, *p* = 0.89, BF_10_ < 0.01).

### Transfer costs and gains

To investigate whether there is a difference in pre-post performance, 2 (Content: fixed, varied) × 2 (Learners’ control: forced, voluntary) × 2 (Block: baseline, transfer block) × 2 (Trial type: repeat, switch) mixed model Frequentists and Bayesian ANOVAs were conducted on both RTs and error rates.

#### RT

As can be seen in Table 4, a significant main effect for block was found. Faster RTs were obtained on the transfer block (*M* = 648 ms, 95% CI [634, 662]) when compared to baseline (*M* = 688 ms, 95% CI [668, 708]). The typical main effect for trial type was also significant with slower RTs on switch (*M* = 735 ms, 95% CI [714, 756]) when compared to repeat (*M* = 602 ms, 95% CI [590, 613]). The corresponding BFs for the two aforementioned main effects indicate strong evidence for the alternative hypothesis.

**Fig. 4:**
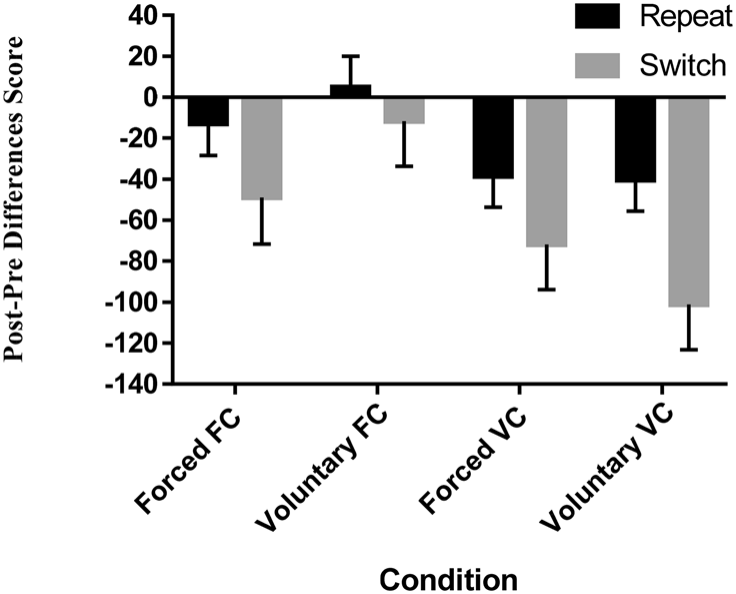
Pre–Post score differences in ms between the groups. Positive values show transfer costs and negative values show transfer gains. Scores are calculated as the difference between baseline and transfer block. Error bars represent standard errors of the mean

The interaction between content and block was significant. As can be seen from Fig. 4, participants in the VC condition show higher training gains (*M* = − 63 ms, 95% CI [− 86, − 39]) in comparison to the FC condition (*M* = − 17 ms, 95% CI [− 41, 6.54]). However, the BF suggested only anecdotal evidence for the alternative hypothesis. No other effect reached significance.

In sum, the results point to a small but significant contribution of varied content training to inducing better transfer gains in task-switching performance.

**Table 4:** Main effects and interactions of the Content × Learners’ control × Block (baseline vs. transfer) × Trial type ANOVA (RTs)

	Statistic	*p* value	Effect size (*η*_p_^2^*)*	BF_10_
Content	*F*(1, 154) = 0.41	0.52	0.003	0.17
Learners’ control	*F*(1, 154) = 0.38	0.54	0.001	0.16
Content × Learners’ control	*F*(1, 154) = 0.14	0.71	0.001	0.21
Block	*F*(1, 154) = 22.57	< 0.001^***^	0.13	> 100
Block × Content	*F*(1, 154) = 7.37	< 0.01^**^	0.04	2.53
Block × Learners’ control	*F*(1, 154) = 0.12	0.72	0.001	0.12
Block × Content × Learners’ control	*F*(1, 154) = 1.63	0.20	0.01	0.30
Trial type	*F*(1, 154) = 326.66	< 0.001^***^	0.68	> 100
Trial type × Content	*F*(1, 154) = 0.30	0.59	0.002	0.14
Trial type × Learners’ control	*F*(1, 154) = 0.42	0.52	0.003	0.16
Trial type × Content × Learners’ control	*F*(1, 154) = 0.93	0.33	0.006	0.07
Block × Trial type	*F*(1, 154) = 16.68	< 0.001^***^	0.10	2.98
Block × Trial type × Content	*F*(1, 154) = 1.34	0.25	0.01	0.22
Block × Trial type × Learners’ control	*F*(1, 154) = 0.05	0.82	0.001	0.17
Block × Trial type × Content × Learners’ control	*F*(1, 154) = 1.73	0.19	0.01	0.48

#### Error data

Differences in error rates revealed the typical switch costs. In addition, the interaction between block and learners’ control was significant (see Table 5). Participants in the voluntary task-switching condition seemed to show slightly higher error rates on the transfer block (*M* = 0.04, 95% CI [0.04, 0.05]) when compared to the forced Task-switching condition (*M* = 0.03, 95% CI [0.02, 0.04]), *t*(156) = 2.64, *p* < 0.05, *d* = 0.28.

**Table 5:** Main effects and interactions of the Content × Learners’ control × Block (baseline vs. transfer) × Trial type ANOVA (error rates)

	Statistic	*p* value	Effect size (*η*_p_^2^*)*	BF_10_
Content	*F*(1, 154) = 0.01	0.91	0.001	0.12
Learners’ control	*F*(1, 154) = 1.98	0.16	0.01	0.29
Trial type	*F*(1, 154) = 23.65	< 0.001^***^	0.13	> 100
Block	*F*(1, 154) = 0.11	0.74	0.001	0.09
Block × Content	*F*(1, 154) = 1.41	0.24	0.01	0.27
Content × Learners’ control	*F*(1, 154) = 0.99	0.32	0.006	0.23
Block × Learners’ control	*F*(1, 154) = 6.30	< 0.05^*^	0.006	2.37
Block × Trial type	*F*(1, 154) = 0.50	0.48	0.003	0.72
Block × Content × Learners’ control	*F*(1, 154) = 0.84	0.36	0.005	< 0.01
Trial type × Content	*F*(1, 154) = 0.70	0.40	0.004	0.18
Trial type × Learners’ control	*F*(1, 154) = 0.70	0.40	0.004	0.19
Trial type × Content × Learners’ control	*F*(1, 154) = 0.80	0.37	0.005	0.25
Block × Trial type × Content	*F*(1, 154) = 0.52	0.47	0.003	0.25
Block × Trial type × Learners’ control	*F*(1, 154) = 0.20	0.65	0.001	0.25
Block × Trial type × Content × Learners’ control	*F*(1, 154) = 1.15	0.28	0.007	< 0.01

### Comparison of transfer costs\gains with Sabah et al. (2018)

An additional aim of the current study is to examine whether the previously observed effect for content variability (Sabah et al. 2018) replicates across studies. Moreover, we intended to explore whether enhanced task interference (the use of bivalent target stimuli in this study as opposed to univalent target stimuli in Sabah et al. 2018) might bear an additional benefit beyond content variability, diminishing the previously observed transfer costs following FC task-switching training condition. To this end, transfer costs and gains between this study and Sabah et al. (2018) were compared. Due to the discrepancies between studies, resulting from the utilization of VTS, only the forced conditions from the current study were considered for analysis. In addition, only the first transfer block from Sabah et al. (2018) was included in analysis, matching exactly the employed transfer block in the current study both in content and task sequence (AA–BB). It is noteworthy that the lack of random assignment between studies results in a possible confound and thus the results of the current set of analyses should be interpreted cautiously.

**Fig. 5:**
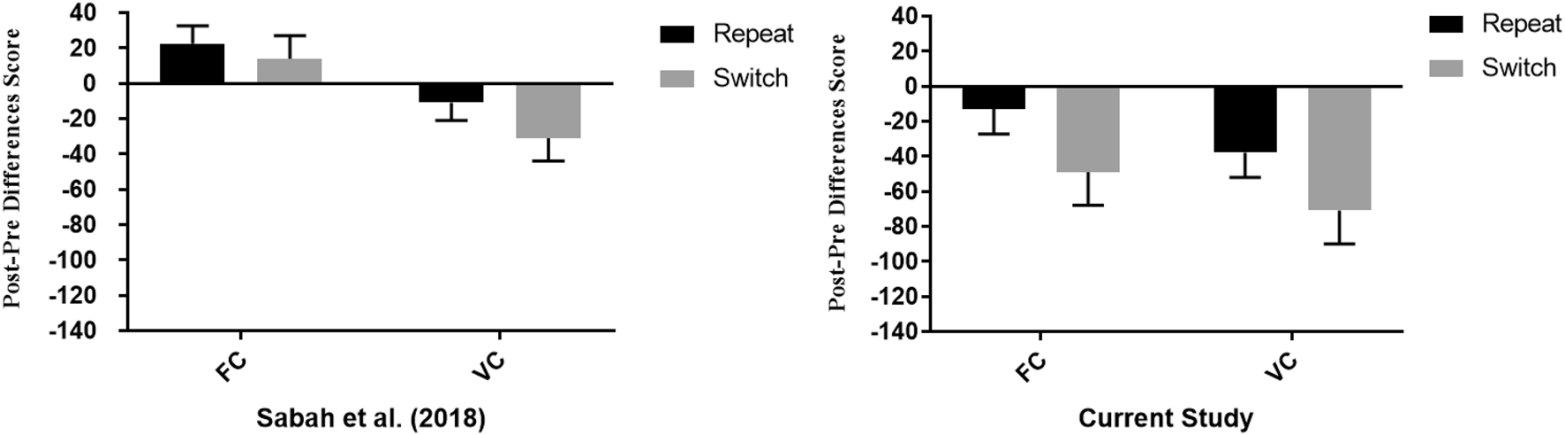
Comparison in transfer costs\gains (difference between baseline and transfer block) between Sabah et al. (2018) and the current study as a function of content variability and trial type. Note that the tasks and sequence in baseline and transfer-block were exactly the same in both studies. Error bars represent standard errors of the mean

For the purpose of the current analysis, study (Current study, Previous study) × Content (FC, VC) × Block (Baseline, Transfer) × Switch (Repeat, Switch) mixed model Frequentists and Bayesian ANOVAs were performed on RTs (See Table 6 and Fig. 5). A significant main effect for block was obtained, with faster RTs observed on the transfer (*M* = 620 ms, 95% CI [610, − 640]) when compared to the baseline block (*M* = 642 ms, 95% CI [627, 657]). The main effect for study was also significant. Overall, slower RTs were observed in the current study (*M* = 673 ms, 95% CI [655, 691]) when compared to Sabah et al. (2018) (*M* = 589 ms, 95% CI [576, 601]). Moreover, the main effect for trial type was significant where faster RTs were obtained on repeat (*M* = 589 ms, 95% CI [580, 599]) as compared to switch trials (*M* = 672 ms, 95% CI [659, 686]).

A significant two-way interaction was found between study and block with higher gains in the current study (*M* = − 43 ms, 95% CI [− 65, − 21]) when compared to Sabah et al. (2018) (*M* = − 1 ms, 95% CI [− 17, 14]). Furthermore, the interaction between block and trial type reached significance. Smaller switch costs were obtained in the transfer block as compared to the baseline block, *t*(230) = 4.22, *p* < 0.001, *d* = 0.27. Importantly, the interaction between content and block was also significant, pointing to higher gains in the VC condition (*M* = − 38 ms, 95% CI [− 56, − 19]) as compared to the FC condition (*M* = − 6 ms, 95% CI [− 25, 12]). This was further supported by the BF, indicating strong support for the alternative hypothesis. In addition, falling in line with the frequentist results, this interaction was not modulated by study, as indicated by the BF for the three-way interaction Study × Content × Block. This last result means that H0 concerning lack of difference between the studies (in this regard) is ~ 32 times more probable than a difference between the studies, given the results (and equal priors for H0 and H1).

Overall, the benefit for content variability to Task-switching performance is successfully replicated, where across studies we see lower costs\higher transfer gains following VC training. Moreover, relative to Sabah et al. (2018), higher transfer gains emerged, possibly due to the utilization of bivalent target stimuli, enhancing task demands.

**Table 6:** Main effects and interactions of Study × Content × Block × Trial type ANOVA (RTs)

	Statistic	*p* value	Effect size (*η*_p_^2^*)*	BF_10_
Study	*F*(1, 227) = 57.26	< 0.001^***^	0.20	> 100
Content	*F*(1, 227) = 1.09	0.30	0.005	0.20
Trial type	*F*(1, 227) = 467.62	< 0.001^***^	0.67	> 100
Block	*F*(1, 227) = 10.78	< 0.01^**^	0.04	3.75
Block × Study	*F*(1, 227) = 9.49	< 0.01^**^	0.04	56
Block × Trial type	*F*(1, 227) = 21.45	< 0.001^***^	0.09	1.11
Block × Content	*F*(1, 227) = 5.59	< 0.05^*^	0.02	13.06
Study × Trial type	*F*(1, 227) = 139.74	< 0.001^***^	0.38	> 100
Study × Content	*F*(1, 227) = 0.13	0.71	0.001	0.20
Content × Trial type	*F*(1, 227) = 0.76	0.38	0.003	0.02
Content × Block × Trial type	*F*(1, 227) = 0.20	0.65	0.001	0.17
Study × Content × Block	*F*(1, 227) = 0.38	0.57	0.001	0.21
Study × Content × Trial type	*F*(1, 227) = 0.004	0.95	0.001	0.19
Study × Block × Trial type	*F*(1, 227) = 3.71	0.05	0.02	0.25
Study × Content × Block × Trial type	*F*(1, 227) = 0.51	0.47	0.002	0.26

## Results: training outcomes on verbal fluency measures

To examine potential training modulation on a structurally dissimilar task of cognitive flexibility, here, verbal fluency, we first calculated a mean score for the total of generated words in the baseline and transfer block. To exclude initial difference between the groups, a one-way Frequentists and Bayesian ANOVAs were performed, pointing to no significant pre-existing difference, *F*(3, 152) = 1.20, *p* = 0.311, *η*_p_^2^ = 0.02, BF_10_ = between the groups. Then, post–pre scores were calculated by subtracting the post from the pre-testing scores. Two-way Frequentists and Bayesian ANOVAs were performed on post–pre scores, entering content (fixed vs. varied) and learners’ control (forced vs. voluntary) as between subject variables. No significant main effect was found for either content or learners’ control, *F*(1,152) = 0.13, *p* = 0.71, *η*_p_^2^ = 0.001, BF_10_ = 0.26, *F*(1,152) = 0.001, *p* = 0.97, *η*_p_^2^ = 0.001, BF_10_ = 0.27. The scores were overall negative (*M* = − 2.15), showing a general practice effect that was not further modulated by group (all BF_10_ < 0.33).

## Discussion

The current study attempted to examine the mutual contribution of variability and learners’ control to training and transfer in short-term Task-switching training. To manipulate content variability, same or different task rules and stimuli were introduced during training. Learners’ control was manipulated by applying either voluntary or forced Task-switching procedure. To enhance task demands during training, bivalent stimuli were used.

Three main findings stand out: First, we replicated the variability effect, found by Sabah et al. (2018), with content variability producing smaller practice effects yet higher transfer gains when compared to the FC condition. In contrast, learners’ control did not induce additional beneficial effect on transfer beyond content variability. Lastly, we compared the present results to those of our former study, in which the exact same tasks during baseline and transfer were performed on univalent stimuli. This comparison showed that the current study yielded more pronounced transfer gains following VC training along with absent transfer costs after FC training. Thus, it is assumed that the enhanced control demands during training (usage of bivalent stimuli) underlie these more favourable outcomes. This latter result however is based on between study comparisons and therefore has to be treated with caution.

As expected, content variability is again found to promote better transfer outcomes in short-term Task-switching training. In line with Sabah et al (2018), disrupting learning by introducing practice variability seems to enhance transfer, with more pronounced benefit on switch when compared to repeat trials. Taken together, these results support the proclaimed notion of “desired difficulty”, denoting the paradoxical nature of learning (Schmidt and Bjork 1992). As such, it is postulated that creating challenging practice conditions can facilitate deeper learning and retention yet withot any observable improvement during training. Despite the encourging pattern of near transfer gains (seen in Task-switching following VC training), no generlization effects were seen on the verbal fluency task. This in turn, falls in line with many recent indications that question the occurance of far transfer in CT (e.g., Dougherty et al. 2016; Melby-Lervåg et al. 2016; Soveri et al. 2017).

With regard to learners’ control, the resultts have failed to support our predictions. No additional transfer benefits beyond content variability were obtained in the voluntary when compared to the forced VC training condition. Consequently, the observed effect for learners’ control in the trainig phase seems to merely reflect the different underlying cognitive process between the two procedures. Similar to Arrington and Logan (2005) our results revealed smaller switch costs in the voluntary when compared to the forced condition. The lack of modulating effect for learners’ control on transfer is quite surprising when considering the existing litreature on learning and motivation, pointing to the contribtion of self-controlled practices to skill acquision across domains (e.g., Chiviacowsky et al. 2012a, b; León et al. 2014; Sanli and Patterson 2013). Hence, self-regulated practice has been suggested to underly effective learning, boosting key motivational componates, such as self-efficacy, higher task engagement and percieved competence (e.g., Bell and Kozlowski 2008; Chiviacowsky et al. 2012a, b; Deci and Ryan 2008; Lewthwaite and Wulf 2012; Ryan and Deci 2000; Tafarodi et al. 1999). In turn, the reason why we did not find any additional beneficial effect of learners’ control might be due to the specific task instructions. That is, while participants were free to choose one of two tasks on each trial, they were also *told* to choose each task equally often but in a random order. This instruction might have increased overall task demands rather than motivation. Furthermore, given that task demands were already high with varied content and bivalent stimuli, performance might have already reached ceiling. The overall higher transfer benefits and lower transfer costs as compared to Sabah et al. (2018) point to this direction.

Directly related to that, another aim of the current study was to explore whether enhancing task demands by utilizing bivalent stimuli might counteract the training costs following FC training condition (Sabah et al. 2018). As such, the results of the current study were compared to Sabah et al. (2018), excluding the voluntary conditions from analysis. The reason for this exclusion was to minimize the influence of excessive procedural variation between the studies when using VTS. As showed by the results, the effect for content variability was preserved across studies with higher training gains obtained here when compared to Sabah et al. (2018). In part, this falls in line with previous findings (Kray and Fehér 2017), showing that enhanced interference demands in Task-switching (as a result of bivalency) leads to improved transfer effects. Nevertheless, unlike these authors, our results suggest that this advantageous outcome is not only restricted to older but also apparent among younger adults. Importantly, in contrast to Sabah et al (2018), no transfer costs in Task-switching performance emerged following FC training in the current study. This suggests on the one hand that higher task engagement by means of increased control demands might have prevented the occurrence of negative transfer. One the other hand, we cannot exclude the possibility of a failure to replicate Sabah et al.’s findings concerning costs. Lastly, it could also be possible that any observed differences between studies might be confounded by task structural differences (i.e., task sequence). In addition to content variability, Sabah et al (2018) introduced another variability manipulation on the deeper level of the task structural configuration, comparing fixed (i.e., alternating runs) with random task sequence. Conversely, in the current study, task sequence in the forced condition was determined by the choices of participants in the VTS conditions (i.e., by the yoking procedure), with task choices only approximating randomness. However, as task structure did not yield any notable effect in Sabah et al.’s (2018) study, such confounding effect seem quite implausible.

A noteworthy unexpected observation relates to the training effect on VSR in the FC and VC condition. Two lines of evidence would have suggested that varied content should increase the rate of voluntary task switching: Fröber and Dreisbach (2017) showed that frequent forced task switching increases cognitive flexibility and thus voluntary task switching (for a review see Dreisbach and Fröber 2019). Additionally, Mayr and Bell (2006) had shown that single stimulus changes (from one trial to the next) invoke higher switch rates than stimulus repetitions. Both of these findings thus suggest that bottom up changes can motivate or otherwise cause voluntary task switching. However, VSR rates in the training blocks clearly point to the opposite direction. Participants switched more often in the FC group and not in the VC group. It seems that at least during repetitive training of the same two tasks, participants tended to switch tasks more frequently, pointing perhaps to the possibility that switching serves as means to prevent boredom (e.g., Inzlicht et al. 2014; see also Jersild 1927). Another interesting observation is the significant increase of VSR over the training blocks, a trend which was observed in both groups. Given that participants were asked to choose tasks equally often and in a random order (which would ideally result in a switch rate of 50%), this increase can in part be explained by the feedback (VSR in %) provided after the end of each block. However, participants in the FC condition had already reached the required 50% in Block 2 but still showed an increasing switch rate over the remaining blocks (see Fig. 2). This further speaks to the idea that participants in the FC may have avoided boredom. Alternatively, and not mutually exclusively, the VSR increase may be an instance of learned industriousness according to which effort is experienced as rewarding, and hence reinforces higher performance (Eisenberger 1992). Future research is clearly needed to further disentangle the mechanisms underlying the differences in switch rates between content conditions.

In sum, the current study provides additional support for the advantage of varied training regimes in short-term Task-switching training, with even more pronounced gains when coupled with increased task demands (i.e. bivalency). Although no notable impact for leaners’ control on transfer was found, the (unpredicted) data pattern of VSR during practice (increasing VSR with increasing practice, higher VSR with fixed than varied content) points in interesting new directions. Future research may therefore address more directly the impact of boredom as an intrinsic modulator on task engagement and training outcome.

## Electronic supplementary material

Below is the link to the electronic supplementary material.

Supplementary file 1 (DOCX 130 kb)

## Data Availability

The datasets generated and analysed during the current study are available in the Regensburg University publication server repository, https://epub.uni-regensburg.de/43045/; 10.5283/epub.43045.

## Footnotes

1 Yoked control means that for every participant in the voluntary switching groups, we created a “forced twin” that received the exact same task sequence. That way we made sure that the respective groups only differ with respect to the voluntary aspect of task choice.
