# Supplementary material for: Enhancing task-demands disrupts learning but enhances transfer gains in short-term task-switching training
Source: Psychol Res. 2020 Apr 18;85(4):1473–87. doi: 10.1007/s00426-020-01335-y (PMC8286950; doi:10.1007/s00426-020-01335-y)
Supplement: Supplementary file 1 — (DOCX 130 kb) [file 426_2020_1335_MOESM1_ESM.docx]

**Appendix A**


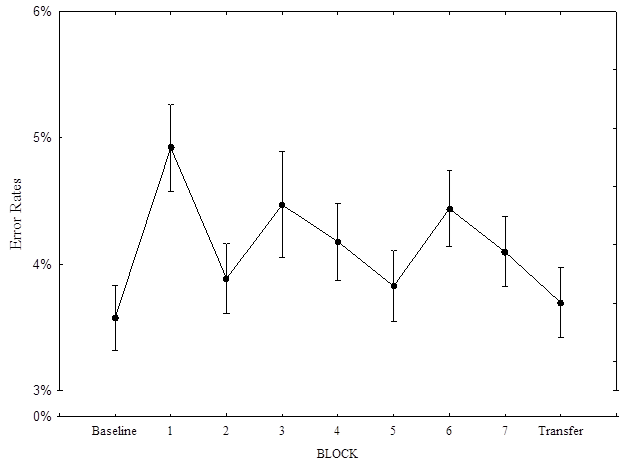


*Fig A1.* Mean error rates along the experimental blocks (collapsed across content and control conditions). Error bars represent standard errors of the mean.


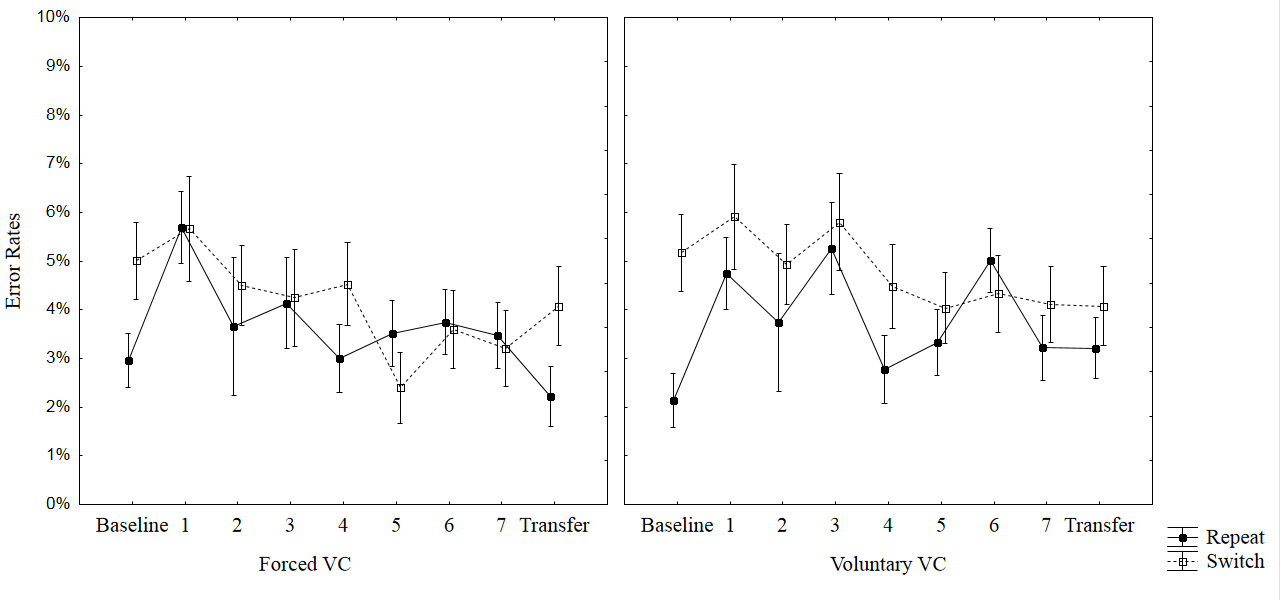


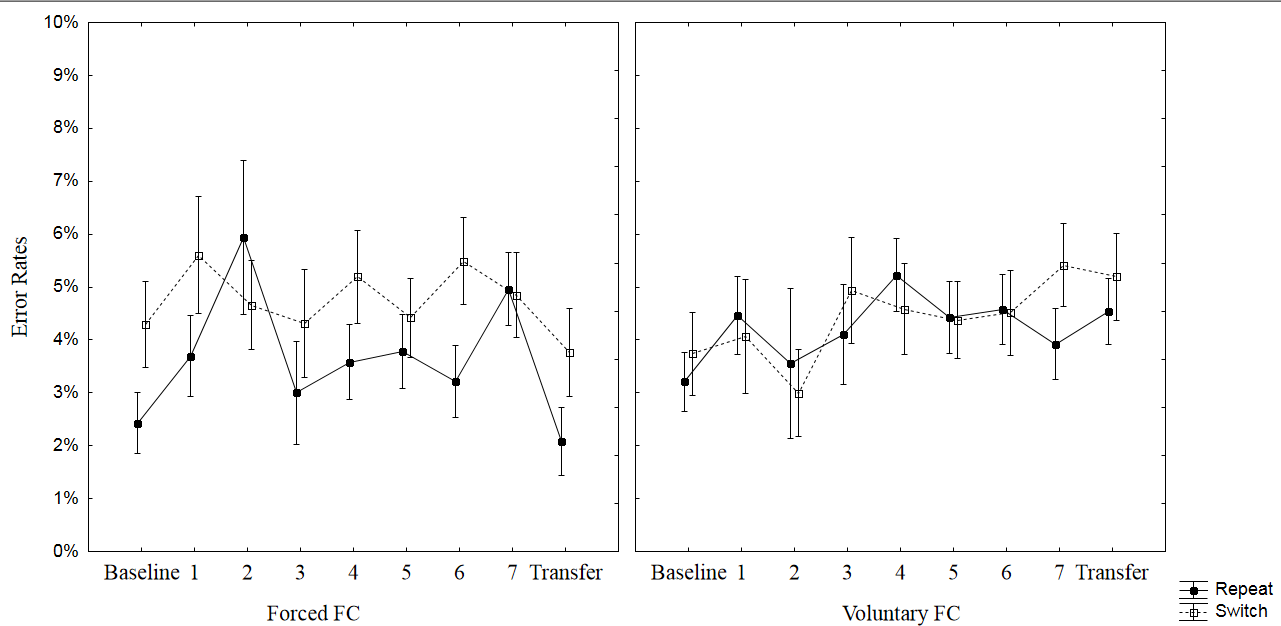


*Fig A2.* Mean error rates along the experimental blocks in the different groups. Error bars represent standard errors of the mean.
